# An observational clinical study of the influence of phacoemulsification on choroidal neovascular membrane activity in age related macular degeneration

**DOI:** 10.1038/s41433-021-01653-4

**Published:** 2021-06-25

**Authors:** H. D. Jeffry Hogg, N. Chung, J. Reed, G. Berrett, M. Pearce, Sandro Di Simplicio

**Affiliations:** 1 The University of Newcastle upon Tyne, Newcastle upon Tyne, Tyne and Wear, NE1 7RU UK; 2 The Newcastle upon Tyne Hospitals NHS Foundation Trust, Newcastle upon Tyne, Tyne and Wear, NE1 4LP UK

**Keywords:** Retinal diseases, Lens diseases, Epidemiology

## Abstract

**Background:**

Thousands of phacoemulsification surgeries are performed on eyes with age-related macular degeneration (AMD) complicated by choroidal neovascular membrane (CNV) in the United Kingdom each year. As populations age this number is expected to rise. Controversy over phacoemulsification’s influence on CNV activity limits the information which clinicians and these patients use to decide on surgery. This observational study aims to resolve this controversy by reporting on intravitreal injection (IVI) frequency as a pragmatic marker of CNV activity in a large cohort.

**Methods:**

A cohort of eyes with AMD complicated by CNV (*n* = 327) that underwent cataract surgery at a single tertiary centre from 2014 to 2019 were identified. These cases were matched by interval since CNV diagnosis at a specified ‘time zero’ within the follow-up of pseudophakic eyes with AMD (*n* = 327). Data concerning demographics, visual acuity (VA) and intravitreal injection frequency before and after ‘time zero’/phacoemulsification were collected.

**Results:**

Following ‘time zero’/phacoemulsification’ the mean reduction in annual IVI frequency was 0.6 injections/year (95% CI 0.4,0.9) and 0.4 injections/year (95% CI 0.1,0.7) in the comparison and phacoemulsification cohorts respectively. The mean VA gain 12 months after phacoemulsification in the intervention cohort was 11.3 (95% CI 9.2,13.4) early treatment of diabetic retinopathy study (ETDRS) letters, with 214 eyes (65.4%) having gained ≥5 ETDRS letters after surgery.

**Conclusions:**

Phacoemulsification has no clinically significant impact on the activity of pre-existent CNV secondary to AMD. Phacoemulsification should be offered to patients with AMD and cataract that limits vision, regardless of CNV activity.

## Introduction

Cataract and age-related macular degeneration (AMD) are the first and third commonest causes of blindness respectively worldwide [1]. They both effect mainly older people and are set to increase further in incidence as populations age [2]. The most recent UK National Ophthalmology Database audit reported on 241,561 phacoemulsification surgeries from 101 centres between September 2018 and August 2019 and found 10.0% of these eyes had AMD [3]. Over this same time period an approximate total of 452,000 phacoemulsification surgeries were performed in England alone. In an American cross-sectional study of 7081 adults over 40 years of age receiving ocular imaging, 4.6% of patients with AMD were of an exudative type, indicating choroidal neovascular membrane (CNV) activity[4]. These numbers suggest thousands of phacoemulsification surgeries are performed on eyes with CNV activity secondary to AMD every year in the UK. This scale of cataract and AMD comorbidity lend meaningful impact to the persistent uncertainty in the literature of phacoemulsification’s potential to exacerbate CNV activity [5, 6]. Aside from prior observational clinical data to support this effect, there is also mechanistic plausibility as choroidal flow has been found to increase following phacoemulsification [7].

Increased CNV activity may translate to more vision loss for patients and an increased requirement for anti-vascular endothelial growth factor (anti-VEGF) intravitreal injections (IVI), which is costly and undesirable [8, 9]. To improve the quality of informed consent for the many phacoemulsification surgeries offered in the context of AMD complicated by CNV activity, these uncertainties in the literature need to be addressed.

A number of methodologies have been applied to provide partial reassurance that phacoemulsification does not appear to have a substantial impact on CNV activity. Many are limited by the size of the cohorts studied, and hence the power to exclude a small effect [10–14]. Other larger studies have focused on the rate of CNV development following phacoemulsification on eyes with early AMD [15, 16]. The use of phakic comparator groups also limits some of these studies as lens opacification can limit the diagnostic accuracy of CNV activity [17]. As such a recent Cochrane review concluded that insufficient evidence exists to describe the influence of phacoemulsification on AMD [18].

This pragmatic observational study aims to quantify the impact of phacoemulsification on the outcomes of greatest impact to patients; visual acuity (VA) and IVI requirement. To address the limitations in the literature a large intervention cohort has been assembled alongside a pseudophakic cohort, matched by time since CNV diagnosis.

## Methods

This is an observational study of patients receiving IVIs of ranibizumab or aflibercept to treat CNV activity secondary to AMD. The electronic medical record at Newcastle Eye Centre, Newcastle upon Tyne, was searched to include all individuals receiving ranibizumab or aflibercept IVIs for CNV activity secondary to AMD between January 2014 and April 2020. Each case was investigated as far back as January 2008 if necessary, to complete eligibility checking and data collection. Data were collected regarding VA, taken to be the best of previously prescribed spectacle or pinhole corrected vision, and anti-VEGF IVI frequency over an 18 month period of interest. Exclusion criteria were <18 months of follow-up, complicated phacoemulsification surgery, documented posterior capsule opacification within the period of interest or active retinal diagnosis other than AMD within this time period. Any procedure besides ranibizumab IVI, aflibercept IVI or routine phacoemulsification within the period of interest also led to exclusion. The first cohort of eyes to be collated was an intervention cohort with inclusion criteria of having had anti-VEGF IVI for AMD prior to phacoemulsification surgery, having >6 months of available follow-up prior to the surgery and >12 months of follow-up after surgery. A second comparison cohort was identified with the aim of identifying eyes that had fully recovered from cataract surgery and were established on anti-VEGF treatment for AMD. To achieve this, inclusion criteria were set for these eyes to have at least 30 months of follow-up after phacoemulsification. The first 12 of these months were intended to exclude any impact phacoemulsification surgery may have had on CNV activity. A 6 month period then followed, where baseline IVI frequency free from the influence of phacoemulsification surgery could be captured. This point, 18 months into the 30 month follow-up period, was taken as ‘time-zero’ to be compared directly to the time of phacoemulsification of eyes in the intervention cohort. The final 12 of the 30 months of follow-up described allowed for observation of any change to IVI frequency or VA independent of phacoemulsification or cataract. Additionally, this second cohort was recruited to individually match the intervention cohort in respect to the time between their first ever IVI and ‘time zero’/phacoemulsification date. This matching aimed to ensure both cohorts were at a comparable stage of AMD progression and were on comparable IVI treatment protocols during the period over which data were collected. Screening for this second comparison cohort was continued until the size of the first cohort was matched. For each eye at ‘time zero’/phacoemulsification patient age, patient gender, eye laterality, VA, time since first anti-VEGF IVI, time since most recent anti-VEGF IVI and number of IVIs in preceding 6 months were noted. Additionally, the number of IVIs received in the following 12 months and the VA ~12 months after ‘time zero’/phacoemulsification were recorded. The exact time interval to this final VA measurement was also recorded.

Change in anti-VEGF frequency after ‘time zero’/phacoemulsification was taken as the primary outcome. To facilitate comparison, the number of IVIs observed in the 6 months prior to ‘time zero’/phacoemulsification was doubled to project an annual IVI rate. Only 6 months of observation prior to phacoemulsification was used as an inclusion criterion to maximise the size of the cohort and therefore the study’s power to detect a small effect. This shorter initial observation period was also selected to give a more precise proxy for pre-operative CNV activity. IVI frequency was used as a pragmatic proxy for CNV activity as besides VA loss, it is the major burden for patients. VA was collected as a secondary outcome, but it is prone to bias from the extent of cataract and retinal atrophy which this retrospective method could not account for. All patients were treated under the same departmental initiation and treat and extend protocols and so any unanticipated confounders of the relationship between CNV activity and IVI frequency were assumed to be randomly distributed. Most continuous baseline variables and outcomes had a non-parametric distribution and so medians and interquartile ranges were adopted for descriptive statistics. Mann–Whitney *U* tests and independent *t* tests were used to compare scalar non-parametric and parametric variables respectively and chi-squared tests were used for categorical comparisons. Baseline characteristics besides VA (*p* < 0.001) were comparable between the two groups (Table 1).

**Table 1:** Characteristics of intervention and comparison eye cohorts immediately prior to phacoemulsification and at ‘time zero’ respectively.

Baseline variable	Intervention cohort *n* = 327	Comparison cohort *n* = 327	*p* value
Age; median (IQR)	83 (79–87)	83 (79–87)	0.579
VA in ETDRS letters; median (IQR)	50 (35–65)	64 (44–72)	<0.001*
Months since first IVI; median (IQR)	32 (21–56)	33 (21–57)	0.886
Months since most recent IVI; median (IQR)	2 (1–5)	2 (1–7)	0.206
Total IVIs in prior 6 months; median (IQR)	2 (1–3)	2 (0–3)	0.3
Female; count (%)	224 (68.5)	230 (70.3)	0.611
Right eyes; count (%)	169 (51.7)	165 (50.5)	0.754

Independent Mann–Whitney *U* tests and Chi-squared tests were used to check for significant differences between the continuous and categorical variables respectively with an * denoting significance following Bonferroni adjustment for the 7 tests (*p* < 0.007 taken as significant). IQR = interquartile range, VA = visual acuity, IVI = intravitreal injection, ETDRS = Early Treatment of Diabetic Retinopathy Study.

Univariable linear regression using the seven baseline variables was performed separately with annual IVI frequency and final VA 12 months after ‘time zero’/phacoemulsification as dependent variables (Table 1). An additional eighth variable, the observed time between ‘time zero’/phacoemulsification and the recording of ‘12 month VA’ was also included for the analysis of VA change. Multivariable linear regression was performed using any variables demonstrating significant predictive value in the univariable analyses to establish the nature and scale of any independent predictors of IVI frequency and VA.

Analysis was conducted using SPSS v.24 (IBM Corporation, New York, USA). Data are reported in accordance with STROBE criteria and approval for this project was granted from The Newcastle upon Tyne Hospitals NHS Foundation Trust Caldicott office (application ID: 7570) [19].

## Results

### Intervention cohort

**Fig. 1:**
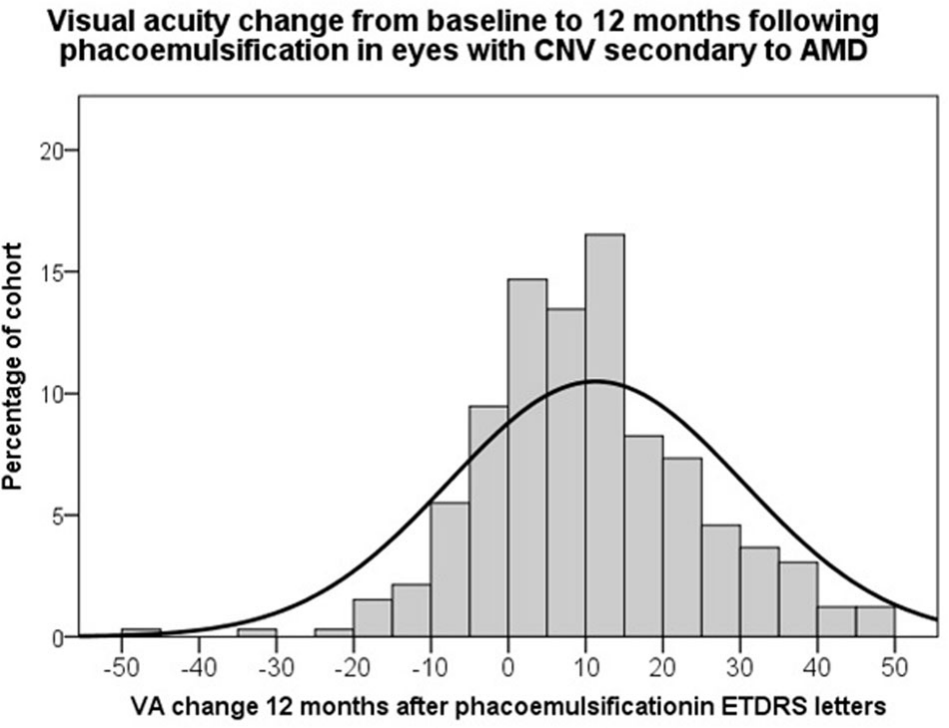
Visual acuity change. Bar and line histogram showing distribution of visual acuity (VA) gain 12 months following phacoemulsification in 327 eyes with prior choroidal neovascular membrane (CNV) activity secondary to age-related macular degeneration (AMD). ETDRS = early treatment of diabetic retinopathy study.

For the intervention cohort, 327 eyes from 302 patients satisfied the eligibility criteria. The median VA 12 months after surgery was 65 (interquartile range 45–75) early treatment of diabetic retinopathy study (ETDRS) letters representing a mean 11.3 (95% confidence interval (CI) 9.2, 13.4) ETDRS letters gained from baseline. Sixty–five eyes in the intervention group (19.8%) had lower VA 12 months following surgery than at baseline and 214 eyes (65.4%) had gained ≥5 ETDRS letters 12 months after surgery (Fig. 1). One hundred eyes (30.6%) in the intervention group had a higher postoperative IVI frequency than at baseline.

### Comparison cohort

**Fig. 2:**
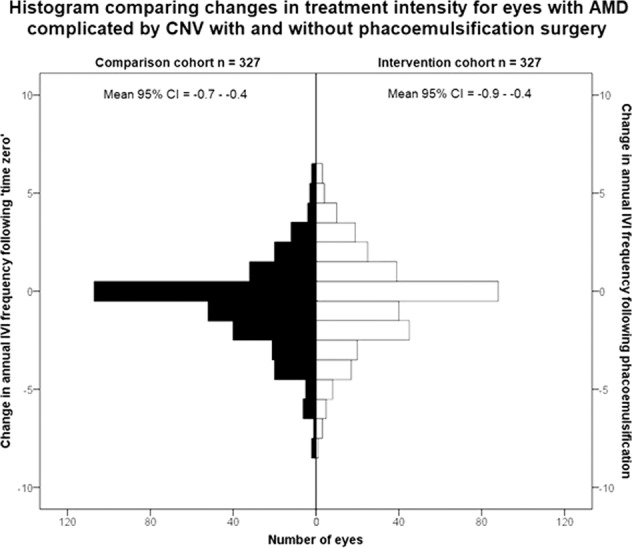
Intravitreal injection frequency change. Histogram showing the equivocal change in annual intravitreal injection (IVI) frequency observed before and after phacoemulsification or ‘time zero’ in the intervention and comparison cohorts respectively. AMD = age-related macular degeneration, CNV = choroidal neovascular membrane.

Three hundred and twenty-seven eyes from 264 patients satisfied matching and eligibility criteria to compose the comparison cohort. These eyes had equivocal baseline characteristics to the intervention cohort besides a significantly higher baseline VA (*p* < 0.001), presumably on account of their lack of cataract (Table 1). The intervention cohort had a mean reduction in annual IVI frequency of 0.4 injections/year (95% CI 0.1,0.7), which was equivocal (*p* = 0.20) to the reduction of 0.6 injections/year (95% CI 0.4,0.9) observed in the comparison group (Fig. 2). Following Bonferroni correction, the number of IVIs observed in the 12 months following phacoemulsification and ‘time zero’ no longer showed significant difference (*p* = 0.026) with medians of 4 (IQR 1–6) and 3 (IQR 0–5), respectively. The comparison cohort lost a mean of 3.9 ETDRS letters (95% CI 2.8,4.9) of VA between baseline and 12 months to reach a median VA of 59 ETDRS letters (IQR 38–72). Both these final (*p* = 0.012) and change (*p* < 0.001) values of VA remained significantly lower than that of the intervention group after Bonferroni correction.

Univariable linear regression of all baseline variables (Table 1), with number of IVIs in the 12 months following ‘time zero’/phacoemlsification as the dependent variable, found that age (*p* = 0.36) and months since first IVI (*p* = 0.79) had no significant predictive power. The remaining five baseline variables underwent multivariable linear regression where gender (*p* = 0.11) and laterality (*p* = 0.65) were also rejected from the model. This left three significant contributors to a model with a total *R*^2^ of 0.45. These variables were membership of the intervention or comparison cohort (b = 0.39 (95% CI 0.07,0.71), *p* = 0.015), months since most recent IVI (*b* = −0.036 (95% CI −0.058, −0.014), *p* = 0.001) and number of IVIs in the 6 months prior to ‘time zero’/phacoemulsification (*b* = 1.04 (95% CI 0.90,1.18), *p* < 0.001). The overall direct and indirect effects contributing to the model were greatest from number of IVIs in the 6 months prior (*R*^2^ = 0.43). Months since most recent IVI (*R*^2^ = 0.23) and membership of the intervention or comparison cohort made smaller, but significant contributions (*R*^2^ = 0.01).

This analysis was repeated with VA 12 months following ‘time zero’/phacoemulsification as the dependent variable, and with the inclusion of the precise time between ‘time zero’/phacoemulsification and the observed ‘12 month’ VA date as an independent variable. Univariable analysis found age (*p* = 0.078), gender (*p* = 0.99), laterality (*p* = 0.44), and months since first IVI (*p* = 0.21) not to have significant predictive power. Multivariable linear regression also rejected number of IVIs in the 6 months prior to ‘time zero’/phacoemulsification (*p* = 0.96) and the precise time between ‘time zero’/phacoemulsification and the observed ‘12 month’ VA date (*p* = 0.13). This left three significant contributors to a model with a total *R*^2^ of 0.57. These variables were membership of the intervention or comparison cohort (*b* = 12.27 (95% CI 9.97,14.6), *p* < 0.001), months since most recent IVI (*b* = −0.24 (95% CI −0.40, −0.08), *p* = 0.003) and baseline VA (*b* = 0.74 (95% CI 0.68,0.80), *p* < 0.001). The overall direct and indirect effects contributing to the model were greatest from baseline VA (*R*^2^ = 0.47). Months since most recent IVI (*R*^2^ = 0.09) and membership of the intervention or comparison cohort also made smaller, but significant, contributions (*R*^2^ = 0.02).

## Discussion

These real-world data show that phacoemulsification does not cause clinically significant exacerbation of established CNV secondary to AMD (Fig. 2). Multivariable regression suggests that phacoemulsification does have a weak independent effect on CNV activity as the 95% confidence interval describes a relative post-operative increase between 0.07 and 0.71 IVIs per year. This small, but clinically irrelevant, effect is consistent with prior work where smaller studies have found no impact from phacoemulsification on CNV but larger studies have suggested a weak influence[11, 17]. Assurance of the clinical irrelevance of any minor influence that phacoemulsification may hold on CNV activity is also provided by the confidence intervals of change in IVI frequency in the intervention and comparison cohorts (Fig. 2). Even at the deleterious limits of these confidence intervals, it seems unlikely clinicians or patients would consider phacoemulsification to be a significant risk in terms of CNV activity. Artificial intelligence tools capable of precisely quantifying volumes of retinal fluid could describe the extent of this subclinical impact through further analysis of observational imaging datasets [20, 21].

In pre-operative discussions patients should be made aware that limits on VA from AMD mean that one-third of eyes will not have clinically significant VA improvement (>5 ETDRS letters) 12 months post-operatively. However, just maintaining VA 12 months after phacoemulsification may represent visual benefit for this group of patients, as the mean VA loss in the comparison group over this time was 3.9 ETDRS letters. The mean 12 month gain of 11 ETDRS letters in the comparison cohort compares poorly to the average of 20 ETDRS letters gained post-operatively from all eyes in the UK National Ophthalmology Database Audit [3]. However, simple VA measurements do not account for the benefit that patients may experience in terms of brightness or glare, but these are hard to measure objectively. Removal of dense cataract will also improve the image quality from both optical coherence tomography and fundoscopy, which will improve the accuracy with which posterior segment disease can be monitored[22].

Whilst this study’s methods avoid some limitations affecting prior studies, there are some persistent biases. Clinicians and patients judged the visual potential of eyes listed for phacoemulsification, to outweigh the risks posed by cataract surgery. This same assurance does not apply to the comparison cohort, which is likely to explain the median 6 ETDRS letters lower VA observed 12 months after ‘time zero’/phacoemulsification in the comparison cohort. It is also possible that this greater representation of eyes with low visual potential in the comparison cohort would prevent further IVI from being recommended, even in the presence of CNV activity. Such an effect may contribute to the slightly lower baseline and final IVI frequencies observed in the comparison cohort, but would likely serve to exaggerate any increase in IVI frequency due to phacoemulsification. Consequently, this affect does not refute CNV activity’s independence from phacoemulsification. As an observational study, completely consistent methods of choosing IVI frequency and VA measurement cannot be assumed. Clinicians practiced under the same departmental protocols throughout the data collection period, but nevertheless variations will have arisen from patient and clinician preferences. The sampling method should have ensured that these variations were distributed randomly between the two cohorts and so should not bias the study’s conclusion.

Establishing the impact of complications in phacoemulsification surgery within the context of AMD would have been an interesting secondary outcome. Only a handful of exclusions were made because of surgical complications and so no meaningful analysis was possible. It seems unlikely that any single centre would have large enough cohorts of eyes with and without CNV experiencing complicated phacoemulsification surgery to comment on this. It may be, that further work involving a subgroup analysis of the UK National Ophthalmology Database could offer an insight into the relative impact of complicated cataract surgery for eyes with CNV. As standardised cataract grading is not routinely performed in the electronic medical record at Newcastle Eye Centre this analysis is also incapable of accounting for the impact of cataract type and density on visual outcome. Whilst it can be assumed that phacoemulsification would not be recommended to patients who were judged to have visually insignificant cataract, it is not possible to comment objectively on what stage a cataract becomes significant in this population. The use of these observational methods was necessitated by the size of cohorts required to exclude small influences on CNV activity, as the resource requirement of a prospective trial on this scale is disproportionate to the clinical value it would add.

In conclusion, clinicians should assure patients with AMD complicated by CNV that phacoemulsification does not significantly impact their need for IVI. They can also expect improvement in VA if they have clinically significant cataract, but should be reminded of the independent limits that AMD imposes on their eyes’ visual potential.

### Summary

#### What was known before

- Prior observational studies have excluded substantial exacerbation of choroidal neovascular membrane activity by phacoemulsification surgery in the context of age-related macular degeneration.

#### What this study adds

- This study shows that although there is a small but significant rise in the frequency of anti-VEGF intravitreal injections for these patients, the effect is far below the threshold of clinical significance and need not impact patients’ decision processes when considering phacoemulsification surgery.
